# Do We Look at a Threatening Person’s Face? The Relationship Between Perception and Observation of Walking Strangers

**DOI:** 10.1177/17470218251406631

**Published:** 2025-11-28

**Authors:** Liam Paul Satchell, Jess Hall, Alex Lee Jones

**Affiliations:** 1 Department of Psychology, University of Winchester, UK; 2 School of Psychology, Sport & Health Sciences, University of Portsmouth, UK; 3 Division of Psychology, De Montfort University, Leicester, Leicestershire, UK; 4 School of Psychology, Swansea University, Swansea, UK

**Keywords:** gait, threat, attractiveness, masculinity-femininity, eyetracking, person perception

## Abstract

Person perception research predominantly focuses on faces as stimuli, and less attention is paid to full-body, moving, stimulus people. Nor how our social perceptions might affect the way we observe unknown people. Here, we present two exploratory studies and a registered third. In Study One, 27 judges observed 12 videos of female targets walking and rated ‘threat’, ‘attractiveness’ and ‘masculinity’. In Study Two, 30 judges observed 22 male and female targets in the same format with the same ratings. The registered Study Three included 48 judges observing the same 22 stimuli. Judges had their attention to target faces recorded with an eyetracker. In all studies time spent observing the targets’ heads decreased over time. In Study One, ratings were associated with time spent observing the targets’ head and these effects changed with observation over time. In Study Two no effects were found. Study Three found weak effects opposing Study One. We find overall meta-evidence of masculinity and attractiveness affecting attention to the faces of unknown others, but the individual study findings were highly inconsistent. Our findings draw attention to the risks of interpreting from an individual study and reflect the benefit of internal registered replications.

## Introduction

In everyday life, we, and the people around us, are dynamic. When encountering strangers in the streets, our first sight of other people usually includes their full body moving in the world. Perceiving risk (i.e. threat) or opportunity from strangers can change the way we behave, such as avoiding, approaching, or ignoring unknown people. These consequences of ‘first impressions’ or ‘zero-acquaintance’ judgements have led many psychologists to study the potential sources of variance in a person that might lead to a particular perception. Typically, this field has adopted the reductionist approach of studying parts of a target person at a time, with the intention of creating a literature that can build up insights to a perception of a complete person. The default approach in the field is to use static photographs of faces as a source of perception (Little et al., 2011; Rhodes, 2006). Further reductionism beyond static photographs of faces is also frequently used, with many studies focusing on only a single aspect of faces, such as face width-to-height ratio (fWHR; Mileva et al., 2014), face colouration (Jones et al., 2016; Jones, 2018), eye colour (Bressan, 2020) and eye size (Geldart et al., 1999), lip fullness (Heidekrueger et al., 2016) or even smaller aspects, such as size of limbal rings in the eyes (Brown & Sacco, 2018; Sacco et al., 2019). Atomizing approaches to studying person perception have been critiqued for creating ‘bubbles’ of literature which may not survive contact with each other (Satchell, 2019). Recent studies have attempted to unify these disparate results by including many studied ‘bubbles’ into a single model, finding that many widely researched aspects of faces (such as fWHR, eye size and skin colouration) contribute far less information to social perception – and in some cases almost nothing – than previously thought (Jaeger & Jones, 2021).

The emphasis on reduction-first approaches to studying person perception has prioritized studying faces and their details. However, we know relatively little about how much time is spent attending to the faces of people when we can observe their whole bodies. In three studies presented here, we ask important questions for the field. First, to what extent are faces focused on when being presented with a whole dynamic body? Second, are perceptions of threat, masculinity-femininity and attractiveness associated with the way in which others are observed? These are important questions for us to understand, given the research priorities in the person perception literature. We present two pilot studies and use a registered report to conduct a third study to address these questions.

### Person Perception

As stated above, much of the existing research into zero-acquaintance perceptions of others relies on static images of faces. This is the case for perceptions of aggression or threat (such as Carré et al., 2009, 2010; Geniole et al., 2015; Hehman et al., 2013), perceptions of masculinity to femininity (Batres et al., 2015; Boothroyd et al., 2007; DeBruine et al., 2006; Holzleitner et al., 2014; Little et al., 2014) and attractiveness (Cooper et al., 2006; Rhodes, 2006; Torrance et al., 2014). One important theoretical reason to study faces is that they are prominent in social interaction; but perhaps a practical reason they have received such focus is that photographs of faces are an efficient type of stimulus to create and use in research. In the most robust research into ‘social perception’ to date, 11,570 participants in 41 different countries rated static images of faces on a range of adjectives, including attractiveness and aggressiveness, efficiently studying a complex phenomenon in a reliable and reproducible way (Jones, DeBruine, et al., 2021)

Beyond faces, the human body has also received research interest, albeit to a lesser extent. For example, studying the effects of female stimulus waist-to-hip ratio on perceptions of attractiveness and health is a well-studied area which uses photographs, drawings or digital manipulations (for an overview see Bovet, 2019). Other elements of the body have been focused on in person perception research. This includes perceptions of photographs of upper bodies (Sell et al., 2009), absorptiometry images (Wang et al., 2018), photographs of whole bodies (Tovée & Cornelissen, 2001) and perceptions of leg length from manipulated photographs of body silhouettes (Sorokowski et al., 2011; Sorokowski & Pawlowski, 2008). However, there is limited research presenting full bodies to participants in a way that reflects the everyday perception of people. Some evidence finds that, in speed dating scenarios where individuals interact, findings from atomized and reduced-stimuli studies seem to replicate and affect person judgements of attractiveness, such as female waist-to-hip ratio, male shoulder-to-hip ratio and height for both sexes (Sidari et al., 2021). For the most part, previous research has presented bodies separately from faces and in a static context.

The body in motion has previously been demonstrated to be important for a range of socially communicative information. Experiments using real CCTV footage of events leading to crimes have found that individuals are able to predict an impending crime from watching how an individual behaves, specifically from ‘distinctive gaits and hand gestures’ (Troscianko et al., 2004, p.93). If a distinctive gait is important for an observer to predict a crime, gait could be used to inform perceptions of threat from a moving individual. The body in motion communicates important information about emotions (Montepare et al., 1987; Roether et al., 2009), identity (Cutting & Kozlowski, 1977; Mather & Murdoch, 1994), vulnerability (Gunns et al., 2002) and trait aggression (Satchell et al., 2018, 2021). Most importantly, the whole body is the most typical way that one encounters others in the world. In the case of observing another person, it is rare to only be exposed to the face of that individual and to have no access to that person’s body, gait and general appearance. Judging the threat, attractiveness, or a host of other social traits of a moving person could therefore be influenced by different aspects of that individual, which have not been as well-studied.

### Eyetracking and Observation of Others

One way to address the importance of faces is to consider how often faces are attended to when observers have the chance to see a whole person. Further, perceptions matter here too, as a face that might be considered more arousing (i.e. a threat or attractive) might draw more attention to the head of a person rather than their body. However, this is not understood well as, similarly to the majority of social perception literature, eye tracking methods in the context of threat detection and person perception have largely focused on photographs of faces. This research typically looks at the recognition and fixation on ‘threatening’ emotional expressions, such as angry faces (e.g. Eastwood et al., 2001) and averted eye gaze (e.g. Fox et al., 2007; Terburg et al., 2011). Some eye tracking research has investigated how individuals visually attend to threatening postures in the whole body. However, these studies also use stimuli that are unrealistic for everyday threat detection, such as mannequins (Gilbert et al., 2011) and exaggerated demonstrations of emotion (Azarian et al., 2016a, 2016b). Most interactions with unknown people do not start from a point of aggressive escalation, from a fighting pose, or whole-body extreme prototypical emotion expression. While studies with methods that use such stimuli contribute to the understanding of how threat is attended to with immediate risks (once someone moves to fight), these studies do not show us how patterns of visual search might affect the evaluation of potential (not imminent) threats.

In other research, eye tracking has been used to investigate what parts of the body are preferentially observed when judging attractiveness. In research using digital manipulations of a full-body photograph of a man in his underwear, female participants spent more time observing the upper body, and their observation time interacted with their perceptions of how attractive they rated the man (Garza et al., 2017). In another study, participants observed images of computer-generated female avatars in their underwear, and it was found that the upper body was preferentially observed compared to the head or lower body (Rodway et al., 2019), although it was not reported if this was affected by how attractive participants found the targets. Work on the observation of clothed targets for attractiveness judgements is less common. One recent example, using the same computer-generated female avatars as Rodway et al. (2019), added different coloured digital dresses to the targets, and they still found preferential observations of the upper body and reported that this was not affected by how attractive participants found the targets (Sidhu et al., 2021).

Overall, there has been relatively limited attention paid to full-body, clothed, moving, real images of target people across the studies using eyetracking to study different perceptual attributes.

#### The Current Studies and Registered Hypotheses

This paper builds on previous eye tracking research in social perception to investigate how perceptions and eye gaze interact when observing full-body stimuli of walking people. The above literatures start from the assumption that the face is an important part of a person that will draw attention, and therefore, we can conduct research on static images of the face as a starting point to build up to the everyday experience of whole-body dynamic people. Whilst it is likely the case that the face attracts attention – and we empirically test this here – there are questions about the extent to which the face is focused on and preferentially observed when viewing a whole-body dynamic person. The face holds many social cues and suggestions of intentionality (Stephenson et al., 2021), but over time, an observer has more time to consider the whole of a body they are seeing, and it is likely that, given a span of time, non-face observation will increase. The extent to which the face is persistently observed during this time might be related to how arousing that targets’ face is. We would suggest that the more ‘arousing’ the face, the more an observer might attend to it. For example, how positively arousing (attractiveness) or negatively arousing (threatening) or simply distinct (masculinity-femininity) a person might seem to be could draw more attention to looking at their face. An attractive or threatening face may seem more worth the attention. The initial appraisal of the face encourages more observation time to better solidify the perception. This dynamic, reciprocal, internal appraisal and attention process is difficult to separate empirically (as these processes happen in fractions of a second). However, we suggest that there is important insight from analyzing how much shared variance there is between the observation of a face as part of a whole-body stimulus and the overall perception (i.e. threat, attractiveness) of that stimulus person. Importantly, we consider how this might change even over a short period of time of observation.

Based on the above reasoning, we make the following three hypotheses for all three studies: (a) The percentage of each second spent observing the head of the target will decrease over time. (b) When judges rate the target people as more threatening, masculine and/or attractive, they will also spend more time observing the targets’ heads. (c) Judges’ ratings will interact with time: targets who are rated more strongly on any domain will have more observations of the head at the start of the videos. We predict this as we expect that the more arousing (on any domain) the judges find the targets, the more the head will draw more attention at the start, as these faces are more distinct.

This registered report describes three studies – two pilot studies which were completed before registering a third. The purpose of this paper is to use a highly powered third study to address inconsistencies in the findings of the two previous studies. To expand, Study One was a proof-of-concept study with a small set of stimuli, demonstrating how perceptions of a person might interact with observation of the targets. As described below, this work found evidence in support of our predictions. Study Two was an attempt to replicate this effect with a larger stimulus set; however, we failed to replicate the findings of Study One. Study One’s findings were theoretically intuitive but based on fewer targets. Study Two’s findings did not support our predictions and previous data, but could be considered a more robust test of the phenomenon. We do not have a consistent message from this previous work, and thus registered a third study, which we approach with an *a priori* defined sample size and defined effect size and use a multi-site approach to collecting data to ensure that Study Three is as robust as possible.

## Study One – Exploratory Study

In this first study, we conducted a proof-of-concept research study. We used a simple design with a small selection of targets, as we were concerned about participants becoming fatigued. We tested our three core hypotheses with an interest in detecting any possible effect in the sample.

### Method

#### Participants

Thirty participants agreed to take part in the first study at a university in the south of the UK. We had no clear power-driven a priori sample size criteria for this study, and we collected a sample that we could afford with the funding available. Those with poor eye tracking calibration (see Procedure, *n* = 2) and those who did not engage with the judgement part of the experiment (*n* = 1) were excluded from final analysis, leaving a sample of 27 participants (*M*_age_ = 26.78, *SD* = 10.02, *N*_Female_ = 20). Participants were paid £5 in shopping vouchers for their time and were recruited from a participant database consisting of members of the public and university students and staff. Participants are henceforth referred to as ‘judges’ to avoid confusion with the targets.

#### Materials

##### Target videos

From a sample of larger male and female stimuli created for other research (see Satchell et al., 2021), we selected all 12 female targets from the stimulus set for use in the first study. We were concerned about how demanding it would be for judges to view a large number of targets and, given that previous research had shown that there is a particularly strong association between gait and trait aggression in females (Satchell et al., 2017), we opted to just use female targets. As we would expect the gait effects to be strongest for female targets, if there was an effect of gait on perception, we should find it in this stimulus set. All 12 targets self-reported being ‘White’ or ‘Caucasian’ (*M*_age_ = 20.58 years, *SD* = 1.78, range = 18–24). All targets were filmed walking on a treadmill wearing standardized clothes (grey or white vest top and black leggings). All videos were 10 s of uninterrupted gait walking towards the camera with targets displaying a neutral facial expression.

##### Areas of Fixation

**Figure 1.**
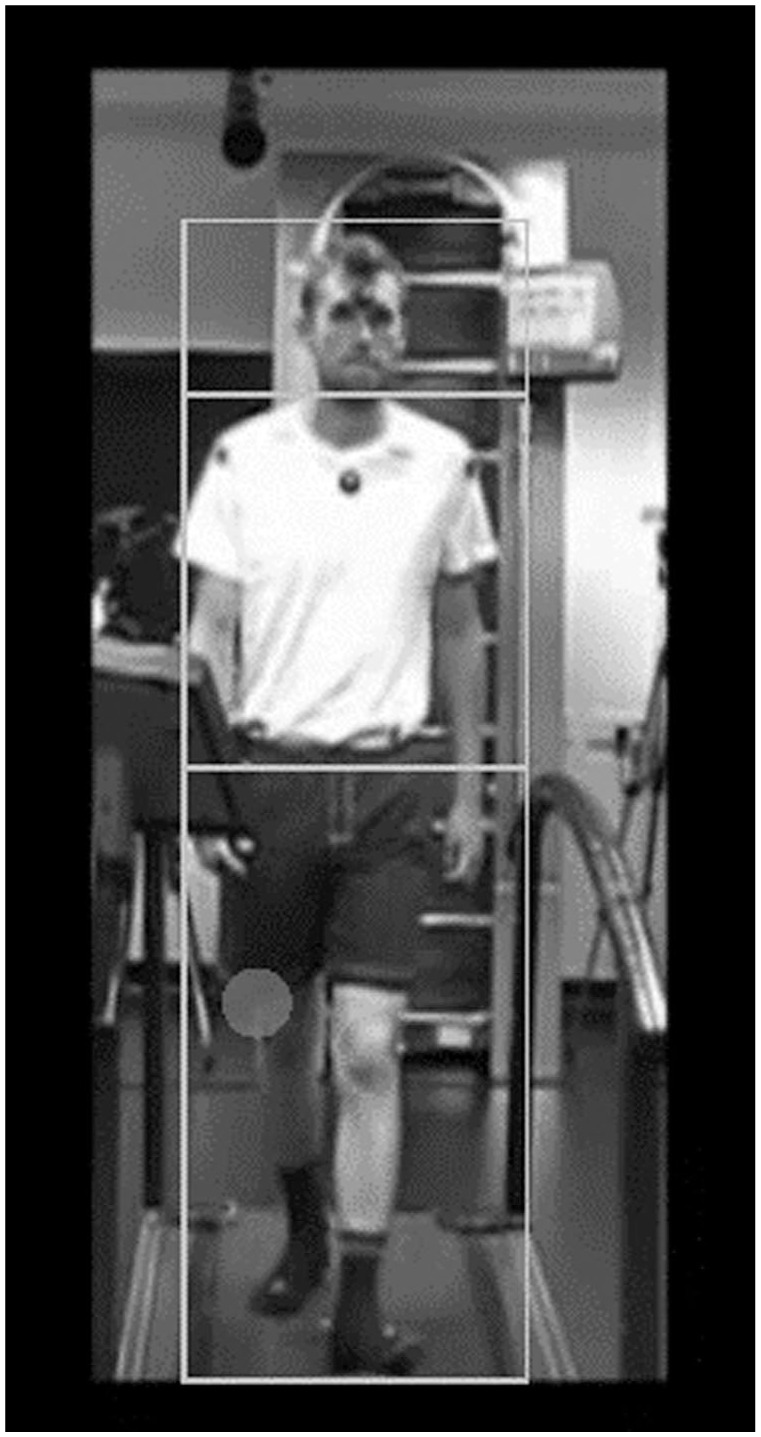
Example of a target image, with interest areas as rectangles. *Note.* In the study, the images were presented in full colour.

The observation of the targets was split into the percentage of each second judges spent studying the three critical areas of the targets: the head (neck and above), the trunk (between neck and hips) and the legs (hips and below). The width of each of these areas was standardized as the width of that target’s shoulders. The areas were consistently placed throughout the trial, and the regions were defined so that they consisted of the key areas throughout the videos. Figure 1 shows an example target with interest areas overlaid. Any observation of the target not within a critical fixation area is considered a non-target observation. In total, the time spent observing the head, trunk, legs and non-target observation is equal to 100%.

#### Procedure

Judges’ eye movements were tracked monocularly at 1,000 Hz with the EyeLink 1,000 (SR Research, Ltd, Osgoode, Canada), using pupil and corneal reflection to detect gaze. Judges placed their heads on a chin-rest at 50 cm from the screen where the targets’ videos were presented. The experiment (the instructions, videos and rating scales) was programmed using experiment builder software, and the same programme is used for all three studies in this paper. Judges first had their eye movements calibrated on the eye tracker. Calibration involved measuring the difference between the expected and actual fixation positions on a nine-point grid presented on the screen. Deviation greater than 0.50° was considered too imprecise, and calibration was repeated until the eye movements were tracked with greater accuracy. If, after repeated calibration, the deviation was still greater than 0.50°, then that judge was excluded from analyses.

After successful calibration, judges were told that they would watch a series of videos and be asked to rate the targets on 1 to 7 Likert scale; *non-threatening-threatening*, *feminine-masculine* and *unattractive-attractive*. This video-then-rating sequence occurred 12 times so that all the judges saw all the targets. Each video was preceded by a drift-checking screen. The presentation order of the targets was randomized for each judge. Rating scales were displayed sequentially on screen, appearing in the same order for each trial: *feminine-masculine*, then *non-threatening- threatening*, followed by *unattractive-attractive*. Participants responded via mouse.

#### Analytic Strategy

**Figure 2.**
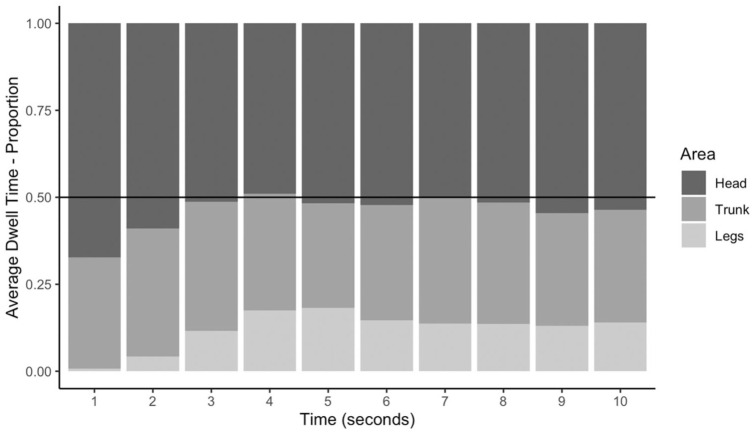
Average dwell time for each area in Study One, binned at each second of viewing time. *Note.* Dwell time is averaged across targets and judges.

All analysis code can be found on the OSF here: https://osf.io/cv7d5/. To test the effects of different perceptions on dwell time per second, we carried out a series of linear mixed models. A simple inspection of the average proportion of dwell time allocated to the head, trunk and legs (see Figure 2) revealed that the head received the majority of the dwell time. Therefore, our analysis was fairly presented by investigating the amount of time per second spent on head observation compared to non-head observation. We focused our analysis on the proportion of dwell time allocated to the head area, predicting this variable from time (seconds 1–10 of viewing) and the perceptual rating of threat, attractiveness and masculinity, as well as the interaction between these variables as fixed effects. We fit three linear mixed models, one for each of the perceptual traits separately. All mixed models included random intercepts for participants and for target stimuli, accounting for variation in both participant and target stimuli ratings.

The time variable was centred on the first second of viewing, casting the time coefficient as the change in dwell time per second with increasing time, and the intercept as the proportion of dwell time in the initial second. Perceptual variables were Z-score standardized within participants.

### Results

All our data for Study One can be found here: https://osf.io/cv7d5/. Overall, the average rating across participants and targets was low on Threat (*M*_*Threat*_ = 2.3, *SD* = 1.4), Masculinity (*M _Masculinity_* = 2.6, *SD* = 1.4) and near-midpoint on Attractiveness (*M_Attractiveness_* = 4.0, *SD* = 1.4).

#### Ratings of Threat

We observed a significant effect of time, *b* = −1.25, *SE* = 0.23, *t*(3193.17) = 16.18, *p* < .001, indicating a 1.25% decrease in dwell time to the head with 1 s increase in time. We also observed a significant effect of perceived threat, *b* = 4.62, *SE* = 1.22, *t*(2598.27) = 3.78, *p* < .001, indicating that a one standard deviation increase of perceived threat was associated with a 4.62% increase of proportion of dwell time on the head in the first second of presentation. There was also a significant interaction between the two variables, *b* = −0.95, *SE* = 0.22, *t*(3193.15) = 4.25, *p* < .001.

**Figure 3.**
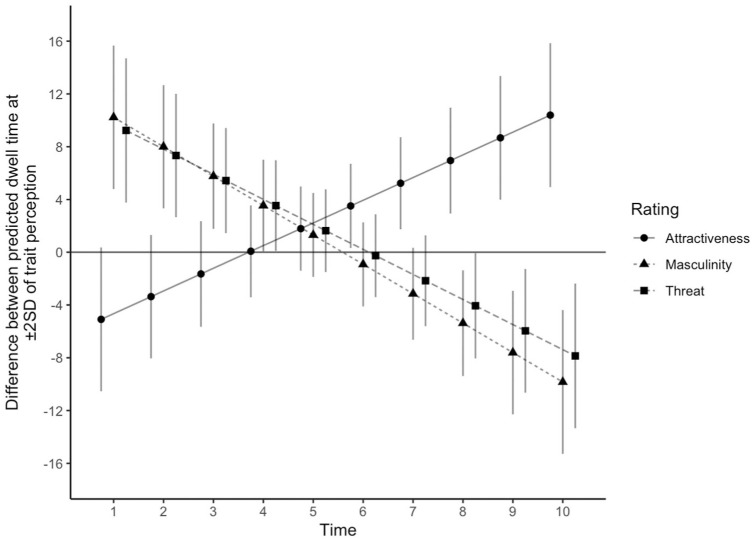
The difference in estimated marginal means of each perception in Study One, evaluated at ±2SD of the rating and at each second of viewing. *Note.* Error bars represent 95% confidence intervals for each of the predicted means.

We examined the estimated marginal means of the model to explore this interaction by using the model to predict dwell time proportion at each of the ten seconds of viewing time for stimuli with a threat rating ±2 SDs about the mean. These estimates are shown in Figure 3. Comparing the high versus low threat scores at each timepoint revealed that the high threat ratings had significantly higher dwell time at seconds one to four (all *p*s < .002), no evidence of a difference at seconds 5 to 7, and significantly lower differences from seconds 8 to 10 (all *p*s < .023).

#### Ratings of Masculinity

For masculinity, we also observed a significant effect of time, *b* = −1.20, *SE* = 0.22, *t*(3312.06) = 5.50, *p* < .001, similarly indicating dwell time decreases by 1.20% with each 1 s increase in viewing time.

We also observed a significant effect of perceived masculinity, *b* = 5.11, *SE* = 1.22, *t*(2285.82) = 4.22, *p* < .001, indicating that a one standard deviation increase in perceived masculinity was associated with a 5.11% increase in the proportion of dwell time on the head in the first second of presentation. There was also a significant interaction between the two variables, *b* = −1.11, *SE* = 0.22, *t*(3312.04) = 5.06, *p* < .001. We used the same approach to explore the interaction as for threat, finding that high masculinity had significantly higher proportions of dwell time for seconds one to four (all *p*s < .022), no significant differences for seconds 5 and 6, and then significantly lower proportions from seconds 7 to 10 (all *p*s < .043).

#### Ratings of Attractiveness

We observed a significant effect of time, *b* = −1.25, *SE* = 0.22, *t*(3312.23) = 5.50, *p* < .001, indicating a 1.25% decrease in dwell time to the head with 1 s increase in viewing time. We also observed a significant effect of perceived attractiveness, *b* = −2.54, *SE* = 1.22, *t*(1.896) = 2.09, *p* = .036, indicating that a one standard deviation increase of perceived attractiveness was associated with a 2.54% decrease in dwell time to the head area in the first second of viewing, the inverse pattern observed with threat and masculinity. There was also a significant interaction between the two variables, *b* = 0.86, *SE* = 0.22, *t*(3312.20) = 3.91, *p* < .001. Exploring this interaction showed that in the first second, high attractiveness was associated with lower dwell time on the head (*p* *=* .036), while no significant differences were observed for seconds two to five. For the remainder of the time, high attractiveness was associated with lower dwell time (all *p*s < .014).

### Study One Summary

In this first proof-of-concept study, we did indeed find effects in line with our predictions. All models supported our first hypothesis that observation of the head decreases over time. We found support for our second prediction, that higher ratings would be associated with longer dwell times on the head, for ratings of threat and masculinity. However, the opposite pattern was observed for attractiveness, with more attractive targets receiving more non-head observation. This suggests that for potential cues to dominance, the head receives more focus, whereas for the affiliative attractiveness rating, whole bodies are observed more often.

In all three ratings, we found interaction effects. The more threatening and masculine a target was perceived to be, there was more dwell time on the head of the target. The less attractive a target, the more dwell time on the head. In all three of these cases, these targets’ heads became less of a focus of dwell time or the videos, going on to have the least observation of their heads by the end.

Overall, here we find evidence that perceptions of targets affect how much attention the head receives when observing a full body of a person in motion. Moreover, this changes over time, with the head becoming less interesting over the observation time of a target, and this interacts with how they perceive that target. However, this study used a very limited stimulus set, only using female targets for our proof of concept. We then attempted to replicate this work with male targets in Study Two.

## Study Two – Exploratory Replication

Study Two was an attempt to replicate Study One using a larger stimulus set. Despite our concerns about participant fatigue in the proof-of-concept study, no participants reported fatigue and the study was completed quickly. Accordingly, our replication attempt includes male and female targets. We maintain the same broad hypotheses, albeit with the updated prediction that the effects for attractiveness are the inverse of the other ratings.

### Method

#### Participants

The sample size heuristic used for this study was to aim for the same sample size as Study One, and so 30 participants we recruited for this study. Due to a data retention error, demographic information about participants was lost, but it was a majority female sample between 18 and 20 years old who were recruited from a university in the East Midlands of the UK. They received course credits in return for participation and were required to have normal or corrected-to-normal vision to participate. Participants are henceforth referred to as ‘judges’ to avoid confusion with the targets.

#### Materials

##### Target Videos

In this study, we used a sample of 11 male and 11 female targets from the existing stimulus set of people walking on treadmills (see Satchell et al., 2021). We wanted an even number of male and female targets, and so, by random number generation, one of the stimuli from Study One does not appear in Study Two. All 22 targets self-reported being ‘White’ or ‘Caucasian’ (*M*_age_ = 20.50 years, *SD* = 1.99, Min = 18, Max = 24). All targets were filmed walking on a treadmill wearing standardized clothes (grey or white vest top and black leggings). All videos were 10 s of uninterrupted gait, walking towards the camera and targets displayed a neutral facial expression.

##### Areas of Fixation

These were devised using the same strategy as in Study One.

#### Procedure

Judges’ eye movements were tracked binocularly at 1,000 Hz with the EyeLink 1,000+ (SR Research, Ltd, Osgoode, Canada), using pupil and corneal reflection to detect gaze. In this study pupil tracked was decided based on participants’ handedness, with right-handed judges being tracked via their right eye and left-handed participants via their left. All other parts of the calibration, experimental programme and procedure were the same as Study One.

#### Analytic Strategy

All analysis code can be found on the OSF here: https://osf.io/cv7d5/. We used the same analytic strategy for Study One in terms of our main hypotheses, analyzing male and female stimuli together. To better replicate Study One, we also conducted the analysis on female targets only. For exploratory analysis, we further conducted the same analysis on male targets only. We also conducted an exploratory and complementary Bayesian analysis to help examine any non-significant effects. This enabled us to make probabilistic statements about the likely magnitude and direction of effects through Bayesian estimation techniques.

### Study Two Results

All our data for Study Two can be found here: https://osf.io/cv7d5/. Overall, the average rating across participants and targets was low on Threat (*M _Threat_* = 2.0, *SD* = 1.4), and near-middle on Masculinity (*M_Masculinity_* = 3.3, *SD* = 1.9) and Attractiveness (*M_Attractiveness_* = 3.5, *SD* = 1.5).

#### Male and Female Targets Together

##### Ratings of Threat

For threat, we observed a significant effect of time, *b* = −1.17, *SE* = 0.17, *t*(5609.93) = 6.93, *p* < .001, indicating that dwell time decreased by 1.17% points with each second of viewing time. There was no significant effect of threat, *b* = −1.26, *SE* = 0.96, *t*(5245.89) = 1.31, *p* = .190, nor evidence of an interaction, *b* = −0.16, *SE* = 0.17, *t*(5609.80) = 0.925, *p* = .355.

##### Ratings of Masculinity

For masculinity, we observed a significant effect of time, *b* = −1.06, *SE* = 0.16, *t*(6267.53) = 6.67, *p* < .001, suggesting that dwell time decreased by 1.06% points with each second of viewing time. There was no significant effect of masculinity, *b* = 1.25, *SE* = 0.99, *t*(1559.98) = 1.26, *p* = .210, nor evidence of an interaction, *b* = 0.25, *SE* = 0.16, *t*(6267.38) = 1.58, *p* = .113.

##### Ratings of Attractiveness

For attractiveness, we observed a significant effect of time, *b* = −1.07, *SE* = 0.16, *t*(6267.26) = 6.67, *p* < .001, suggesting that dwell time decreased by 1.06% points with each second of viewing time. There was no significant effect of attractiveness, *b* = −0.20, *SE* = 0.90, *t*(5807.75) = 0.23, *p* = .819, nor evidence of an interaction, *b* = 0.06, *SE* = 0.16, *t*(6267.12) = 0.38, *p* = .702.

#### Female Targets Only

We repeated the same analysis as above, but using only the data for female targets.

##### Ratings of Threat

For females only, we observed a significant effect of time, *b* = −1.34, *SE* = 0.25, *t*(2786.24) = 5.27, *p* < .001. There was again no significant effect of threat, *b* = 0.78, *SE* = 1.69, *t*(2795.94) = 0.46, *p* = .645, nor evidence of an interaction, *b* = −0.35, *SE* = 0.30, *t*(2786.08) = 1.14, *p* = .253.

##### Ratings of Masculinity

For masculinity, there was again a significant effect of time, *b* = −1.22 *SE* = 0.36, *t*(3113.18) = 3.40, *p* < .001. There was no significant effect of masculinity, *b* = 1.60, *SE* = 1.99, *t*(3139.76) = 0.80, *p* = .421, nor evidence of an interaction, *b* = −0.03, *SE* = 0.36 *t*(3113.04) = 0.08, *p* = .939.

##### Ratings of Attractiveness

For attractiveness, we observed a significant effect of time as before, *b* = −1.23, *SE* = 0.23, *t*(3113.19) = 5.36, *p* < .001. There was no significant effect of attractiveness, *b* = −1.73, *SE* = 1.32, *t*(2895.78) = 1.31, *p* = .191, nor evidence of an interaction, *b* = 0.13, *SE* = 0.23, *t*(3112.98) = 0.56, *p* = .578.

#### Male Targets Only

We also examined, as an exploratory additional test, the perceptions of male targets alone, repeating the above analysis using only data gathered from male targets.

##### Ratings of Threat

For males only, we observed a significant effect of time, *b* = −1.09, *SE* = 0.25, *t*(2796.02) = 4.29, *p* < .001. There was a significant effect of threat, *b* = −3.19, *SE* = 1.27, *t*(2304.96) = 2.50, *p* = .012, but no evidence of an interaction, *b* = −0.12, *SE* = 0.22, *t*(2796.06) = 0.53, *p* = .594.

##### Ratings of Masculinity

For masculinity, there was again a significant effect of time, *b* = −1.55, *SE* = 0.36, *t*(3123.04) = 4.27, *p* < .001. There was no significant effect of masculinity, *b* = 0.32, *SE* = 2.01, *t*(3114.86) = 0.16, *p* = .874, but there was evidence of an interaction, *b* = 0.79, *SE* = 0.36, *t*(3123.16) = 2.19, *p* = .02, indicating that dwell times on the head increased with longer viewing time and higher levels of perceived masculinity.

##### Ratings of Attractiveness

For attractiveness, we observed a significant effect of time as before, *b* = −0.91, *SE* = 0.24, *t*(3123.08) = 3.78, *p* < .001. There was no significant effect of attractiveness, *b* = 0.85, *SE* = 1.37, *t*(2730.22) = 0.62, *p* = .535, nor evidence of an interaction, *b* = 0.09, *SE* = 0.24, *t*(3123.02) = 0.37, *p* = .712.

### Exploratory Analysis – Bayesian Estimation

The non-significant results of both the main effects of rating and their interactions with time, across all three perceptions, are uninformative. To extract more information from these null results, we used Bayesian approaches to re-fit the main mixed models used above, to examine the posterior distributions for each of the three coefficients (time, rating and time by rating). Working with the posterior distribution, rather than a point estimate, has several distinct advantages, especially for non-significant results. For example, in addition to estimating the mean and variability of the effect alongside the 95% credible interval (that is, the region with 95% probability the effect is in), we are able to compute the probability of direction (Makowski et al., 2019) – how likely the effect is to be positive or negative – as well as how much of the posterior might fall within a ‘region of practical equivalence’ (ROPE), an span of estimates that could be considered practically equivalent to no effect (Jones, Jaeger, et al., 2021; Kruschke, 2018). We set a ROPE here of ±0.50, which is a conservative region. In the context of the dependent measure, this means we consider effects as small as half a percentage point of dwell time equivalent to no effect. These offer more insight into the non-significant effects observed. Bayesian analysis requires the specification of prior distributions on the parameters. We opted here to simply leave these at their defaults of a uniform prior, which is mathematically equivalent to maximum-likelihood estimation. That is, the prior has essentially zero influence on the estimates as it carries no probabilistic information. We fit all models using the ‘brms’ package in R (Bürkner, 2018).

**Figure 4.**
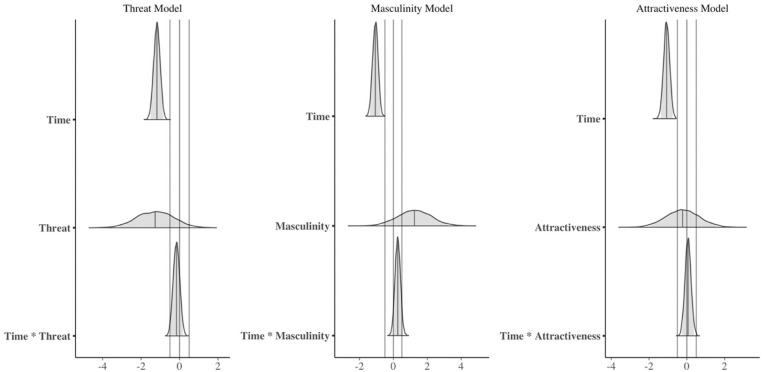
Posterior distributions of each model coefficient in Study Two Central vertical line represents zero; the lines of either size indicate the ROPE region of ± 0.50 (half a percentage point).

The results of the three mixed models are shown in Table 1 and depicted graphically in Figure 4. We focus the discussion on the main effects of rating and the interaction, given that the time results are in line with the frequentist results obtained earlier. For the threat model, the probability that the main effect is negative (i.e. increased threat leads to lower dwell time on the head) is approximately 91%, with around one-fifth of the posterior falling within the ROPE (18%). While this suggests the effect is in the hypothesized direction, the general magnitude is probably small, around 3% points, as indicated by the lower 95% credible interval. However, while the interaction with time is likely to be negative (*p* = .820), much of the posterior falls within the null region (98%).

**Table 1.** Posterior Summaries From the Three Rating Models.

Rating	Parameter	Posterior mean	Posterior SD	95% Lower	95% Upper	*p*(parameter| data) < 0	*p*(parameter| data) null
Threat	Time	−1.178	0.171	−1.517	−0.845	1.000	.000
	Threat	−1.286	0.980	−3.230	0.600	.906	.032
	Time * Threat	−0.157	0.173	−0.496	0.181	.821	.309
Masculinity	Time	−1.066	0.159	−1.379	−0.757	1.000	.000
	Masculinity	1.242	1.003	−0.724	3.227	.109	.036
	Time * Masculinity	0.253	0.164	−0.072	0.570	.064	.154
Attractiveness	Time	−1.065	0.158	−1.380	−0.756	1.000	.000
	Attractiveness	−0.193	0.905	−1.959	1.567	.584	.086
	Time * Attractiveness	0.059	0.161	−0.264	0.373	.350	.423

For masculinity, the probability of direction for the main effect suggests that the effect is likely to be positive, indicating a longer dwell time on the head of more masculine stimuli, and also that the effect is similarly likely to fall within the null region as for threat (19%). While the interaction is likely to be positive (95%), in opposition to the effects observed in Study One, most of the posterior falls within the null region (94%). For both effects, their absolute magnitude is still most probably small, as indicated by the 95% credible intervals.

Finally, for attractiveness, the probability of the direction is relatively uncertain with a negative bias (60%), with around 40% the posterior mass within the null region. The interaction with time falls almost entirely within the null region (99%).

The Bayesian analysis presented here offers further information about the data. Across the three ratings, it is probable that the main effects of threat and masculinity are negative and positive, respectively; attractiveness is much less certain. However, the interactions with time for all three ratings, given the data, are most probably null – when 95% of the posterior mass falls within a null region, the parameter can most likely be rejected (Kruschke, 2018).

### Study Two Summary

Study Two was, methodologically, a more complete test of the target phenomenon. In everyday life, we do not just encounter female approachers, so having male and female targets presented together was an important update to the study. However, Study Two failed to replicate any of the ratings-based effects in the study, even when we looked at female targets by themselves in the data. Other than the increased target numbers and different location of data collection, there were limited differences between the designs. The consistency of the effect of time suggests that some elements of Study One were robust; however, given the theoretical intuitiveness of the Study One results for perception data, it was somewhat surprising that these other effects did not replicate. Bayesian analyses suggested that the direction of the threat and masculinity effects are most likely negative and positive, but not conclusively so. The interaction effects are most likely null. The analyses also confirmed the relatively small size of the effects. Overall, Study Two was a failure to replicate the effects of Study One.

## Study Three – Registered Replication

The results of Study Two and Study One lead to a contradiction in findings. Whilst we consider Study Two’s methodology to be an improvement, the difference between the studies is not so great that we can dismiss Study One’s findings from this failure to replicate. Therefore, we needed more data. We registered a third Study which collected data from two sites based on clear *a priori* defined power considerations to once again test our hypotheses.

## Study Three Method

### Participants

#### Power Analysis

##### Smallest Effect Size of Interest and Model Specification

We based our Smallest Effect Size of Interest (SESOI) on a careful review of the literature. Perceptions of social traits from faces have been estimated to occur in as little as 50 ms (Borkenau et al., 2009), and perceptions of aggressiveness appear to be consistent after just 100 ms exposure to faces (Willis & Todorov, 2006). Other studies demonstrate that perceptions of dominance of faces and bodies are accurate at around 94 ms (Rule et al., 2012), and do not alter much with increased viewing time.

Our main theoretical test of interest here is the main effect of threat. We predicted that higher perceptions of higher threat should, in the first second of viewing a face, lead to shifts away from the face to the body. Based on previous estimates of how quickly this can be appraised, we sought to power our study to detect an SESOI estimate of approximately −10. To place this effect in context, our model specification is of the form:

$$\begin{array}{l} \mathrm{Dwell}\;\mathrm{Time}=(\beta 0+\beta_{\mathrm{participant}}+\beta_{\mathrm{Target}})+\mathrm{Threat}*\beta 1+\\ \;\;\;\;\;\;\;\;\;\;\;\;\;\;\;\;\;\;\;\;\;\;\;\;\;\;\;\mathrm{Time}*\beta 2+\mathrm{Time}*\mathrm{Threat}*\beta 3 \end{array}$$

where *dwell time* is measured in percentage of a given second spent fixated on the head/face of a target. The *time* coefficient is represented as an offset from the first second (each target being displayed for ten seconds, e.g. 0, 1, 2. . .), and *threat* is a z-score standardized rating of the aggression of the target provided by each participant. As we centre time on zero (second one of viewing) and standardize threat, the intercept represents the average dwell time on a face of average threat in the first second of viewing. Thus, the effect of threat, β1, when set to our SESOI of −10 (10% of a second, or 100 ms) represents an effect such that as threat increases, dwell time will be lower in the first second, and this theoretically should decrease to around a tenth of a second, or 100 ms.

It is worth noting that this effect of threat depends on the value of the intercept. The value of the intercept in Study 2 is 39 (390 ms), and in Study 1, 56 (560 ms). Thus, a SESOI of −10 allowed us to detect effects as small as a 100 ms dwell time for high threat faces. Consider that, with an intercept of 39, and a face with a perceived threat score 2 SDs above the mean, dwell time should be around 190 ms (39 + (2 * −10)), and a larger intercept would require an even larger effect of threat to reach the theoretically important value of 100 ms.

##### Sample Size

To complete a power calculation for our model specification, we took a simulation-based approach, generating 500 repeated datasets for each of a series of candidate sample sizes and assessing the proportion in which statistical significance was achieved. To generate datasets, we took the following steps. For threat ratings, we sampled normally distributed data representing ratings for 22 targets (the same as used in the previous studies), one distribution per participant. The time variable was the set of numbers from zero to nine, representing the 10 s of viewing time centred on the first second. The interaction was simply the two variables multiplied by one another. Random intercepts for both targets and participants were sampled as normal distributions with mean zero and standard deviation of 5 and 10, respectively. Dwell time was thus generated as a linear combination of the above variables, and normally distributed noise was added to it from a distribution with a mean of zero and standard deviation of 20. The standard deviations of the noise and random intercepts were informed by the estimates from the model fitting in Study Two. It is worth noting that larger values here made for a more conservative, less confident (i.e. the data is noisier) approach.

We also used the estimates of the intercept (39.83), time coefficient (−1.18) and interaction coefficient (−0.15) from Study Two to generate the data. The time coefficient was set to our SESOI. Candidate sample sizes were set to 30 through 90 in steps of ten participants. Simulations were carried out in R and our code is available here (https://osf.io/cv7d5/ ).

For a threat SESOI of −10, we have 100% power to detect an effect with 30 participants. As another methodological difference between studies (albeit one we do not expect to meaningfully impact the results) was a difference in study location, we aimed to collect 30 participants at two sites: one in the south and one in the midlands of the UK, giving an overall sample of 60 participants but also allowing us more than suitable power to meaningfully look within two samples.

#### Recruited Sample Details

Participants were recruited from two Universities in the UK, one from the South (*n* = 30) and one from the East Midlands (*n* = 30), and are treated as combined for further analysis (*n*_Female_ = 48, *n*_Male_ = 12, *M*_age_ = 24.3, *SD* = 8.5, Miles test eye dominance *n*_RightEye_ = 48, *n*_LeftEye_ = 38, *n*_missing_ = 1). Both sites used the same recruitment method, the same eyetracking hardware (Eyelink 1,000+), and the same experimental software. Participants received course credits in return for participation and were required to have normal or corrected-to-normal vision to participate. Participants with low calibration values or distractions in the experimental setting were removed, leaving a total *N* = 48 (with *n* = 20 and *n* = 28 from each site, with no location-specific data losses). Participants are henceforth referred to as ‘judges’ to avoid confusion with the targets.

#### Materials

##### Target Videos

The same videos as Study Two were used here, as we conducted that study again. This stimulus set contains the same videos as Study One, so if the effects from that study can be replicated, this is the best chance.

##### Areas of Fixation

These were devised using the same information as in Study One.

#### Procedure

##### Eye Tracking

Judges’ eye movements were tracked monocularly at 1,000 Hz with the EyeLink 1,000 and 1,000+ (SR Research, Ltd, Osgoode, Canada) on each site, respectively, using the desktop mount and chin and head rest. We used the same protocol and programme as Study One and Two, using pupil and corneal reflection to detect gaze. Judges placed their heads on a chin-rest at 50 cm from the screen where the targets’ videos were presented. The pupil tracked was decided based on participants’ dominant sighting eye, established using a version of the Miles test (Miles, 1929) in which participants’ hands are brought together to form an aperture for viewing a distant target, and each eye is closed in alternation to establish which is being used. Calibration involved measuring the difference between the expected and actual fixation positions on a 9-point grid presented on the screen. Deviation greater than 0.50° was considered too imprecise, and calibration was repeated until the eye movements were tracked with greater accuracy. A validation procedure using 10 fixation points followed the calibration.

As with the previous studies, each video was preceded by a drift-checking screen (fixation dot). A repeated failure to fixate on the dot triggered recalibration. The presentation order of the targets was randomized for each judge. Rating scales were displayed sequentially on screen, appearing in the same order for each trial: *feminine-masculine*, then *non-threatening-threatening*, followed by *unattractive-attractive*. Judges responded via mouse.

#### Analytic Strategy

We followed a similar analytic strategy in Study Three as in the previous studies. Each rating was tested in its own linear mixed model, with a main effect of time, trait, and their interaction. Random intercepts for participant and stimuli were included. Time was centred on the first second of viewing, and the three rating variables were *z*-score standardized within participants. If we observed non-significant effects, we implemented Bayesian mixed model approaches to draw further inferences about the data.

### Study Three Results

All our data for Study Three can be found here: https://osf.io/cv7d5/. Overall, the average rating across participants and targets for Threat was similar to that of Study 2, being quite low, (*M_Threat_* *=* 2.29, *SD* = 1.49), and similarly near the middle for Masculinity (*M_Masculinity_* = 3.46, *SD* = 1.98), as well as Attractiveness (*M_Attractiveness_* = 3.51, *SD* = 1.45).

#### Main Registered Analysis – Male and Female Targets Together

##### Ratings of Threat

For threat, we again observed a significant effect of time, *b* = −1.70, *SE* = 0.11, *t*(12454.19) = 14.86, *p* < .001, indicating that dwell time decreased by 1.70% points with each second of viewing time. There was no significant effect of threat, *b* = −0.88, *SE* = 0.66, *t*(11542.08) = 1.33, *p* = .185, nor evidence of an interaction, *b* = 0.10, *SE* = 0.12, *t*(12451.21) = 0.86, *p* = .387.

##### Ratings of Masculinity

For masculinity, we observed a significant effect of time, *b* = −1.70, *SE* = 0.12, *t*(12453.51) = 14.86, *p* < .001, suggesting that dwell time decreased by 1.70% points with each second of viewing time. As in Study 2, and with a similar magnitude, there was no significant effect of masculinity, *b* = 1.23, *SE* = 0.76, *t*(640) = 1.62, *p* = .106, nor evidence of an interaction, *b* = −0.10, *SE* = 0.12, *t*(12450.45) = 086, *p* = .391.

##### Ratings of Attractiveness

For attractiveness, we observed a significant effect of time, *b* = −1.70, *SE* = 0.11, *t*(12454.25) = 14.86, *p* < .001, suggesting that dwell time decreased by 1.70% points with each second of viewing time. In contrast to Study 2, there was a significant effect of attractiveness, *b* = −1.82, *SE* = 0.63, *t*(12016.34) = 2.90, *p* = .004, suggesting higher attractiveness ratings were associated with a reduced dwell time on the head. There was no evidence of an interaction, *b* = 0.17, *SE* = 0.111, *t*(12451.23) = 1.50, *p* = .133.

#### Female Targets Only

##### Ratings of Threat

**Figure 5.**
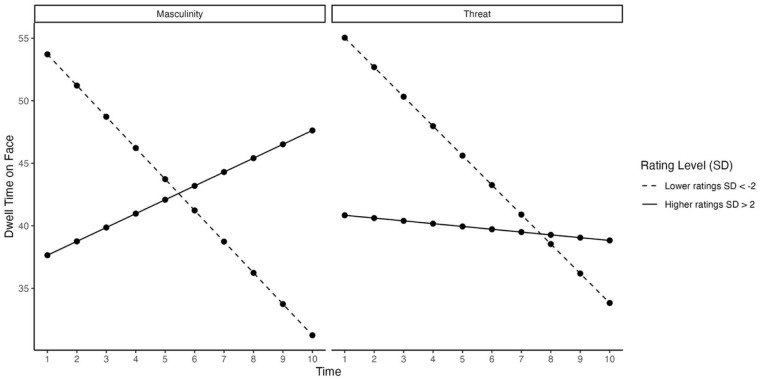
The marginal predictions of dwell time in Study Three at high (+2 SD) and low (−2 SD) rating levels for the statistically significant interactions with female-only targets.

For females only, we observed a significant effect of time, *b* = −1.25, *SE* = 0.18, *t*(5643.53) = 6.82, *p* < .001. There was a significant effect of threat, *b* = −3.62, *SE* = 1.10, *t*(5642.07) = 3.28, *p* = .001, indicating increased threat from female targets was associated with lower dwell times on the head. In addition, there was a significant interaction, *b* = 0.55, *SE* = 0.20, *t*(5641.87) = 2.72, *p* = .007. We explored this interaction in the same way as in previous analyses, by estimating the marginal means of the model, predicting dwell time for each of the 10 s of viewing time, for a stimulus with a threat rating ±2 SD about the mean (shown in Fig 5). Comparing high to low threat scores at each second showed that, at seconds zero to four, low levels of threat had significantly higher dwell times than higher levels, all *p*s < .026, while at seconds five to nine, these differences were non-significant (all *ps* > .176). This suggests that the impact of threat for dwelling on the face of female targets is limited to the initial seconds of an observation. Notably, the effect of high threat was relatively constant, while low threat dropped across time,

##### Ratings of Masculinity

For masculinity, there was a significant effect of time, *b* = −0.69, *SE* = 0.32, *t*(6184.86) = 2.16, *p* = .031, as well as masculinity, *b* = −4.02, *SE* = 1.79, *t*(6062.37) = 2.24, *p* = .025, suggesting increased time and perceived masculinity led to lower dwell times on the head. Moreover, there was an interaction between these variables, *b* = 0.90, *SE* = 0.33, *t*(6184.36) = 2.77, *p* = .006. Different from the effect of threat, high perceptions of masculinity showed significant differences at seconds zero and one (*p*s < .043), non-significant differences at seconds two to seven (*p*s > .093), and further differences at seconds eight and nine, *p*s < .039. These differences had a notably different pattern to that of threat, in that while the low masculinity perceptions led to decreasing fixations on the head with increasing time, high masculinity perceptions led to an increase over time (see Figure 5).

##### Ratings of Attractiveness

For attractiveness, we observed a significant effect of time as before, *b* = −1.51, *SE* = 0.16, *t*(6187.99) = 9.20, *p* < .001. There was a significant effect of attractiveness, *b* = −2.44, *SE* = 0.91, *t*(5874.98) = 2.69, *p* = .007, suggesting that increased perceptions of attractiveness led to lower dwell times on the head. There was no evidence of an interaction, *b* = 0.22, *SE* = 0.16, *t*(6183.96) = 1.36, *p* = .174.

#### Male Targets Only

##### Ratings of Threat

For male targets only, we observed a significant effect of time, *b* = −2.03, *SE* = 0.18, *t*(5675.80) = 11.33, *p* < .001, suggesting a sharper decrease in dwell time for male targets in this condition. There was no evidence of an effect of threat, *b* = −0.62, *SE* = 0.92, *t*(4724.13) = 0.67, *p* = .500, nor an interaction, *b* = 0.04, *SE* = 0.17, *t*(5674.59) = 0.25, *p* = .800.

##### Ratings of Masculinity

For masculinity, there was again a significant effect of time, *b* = −1.99 *SE* = 0.30, *t*(6217.99) = 6.60, *p* < .001. There was no significant effect of masculinity, *b* = 1.08, *SE* = 1.63, *t*(6113.19) = 0.66, *p* = .506, nor an interaction, in contrast to the exploratory analysis of Study 2, *b* = 0.06, *SE* = 0.30, *t*(6217.50) = 0.20, *p* = .845.

##### Ratings of Attractiveness

For attractiveness, we observed a significant effect of time as before, *b* = −1.94, *SE* = 0.17, *t*(6219.25) = 11.75, *p* < .001. There was no significant effect of attractiveness, *b* = −1.36, *SE* = 0.91, *t*(6032.23) = 1.49, *p* = .135, nor evidence of an interaction, *b* = 0.04, *SE* = 0.17, *t*(6217.21) = 0.21, *p* = .830.

### Bayesian Estimation

**Figure 6.**
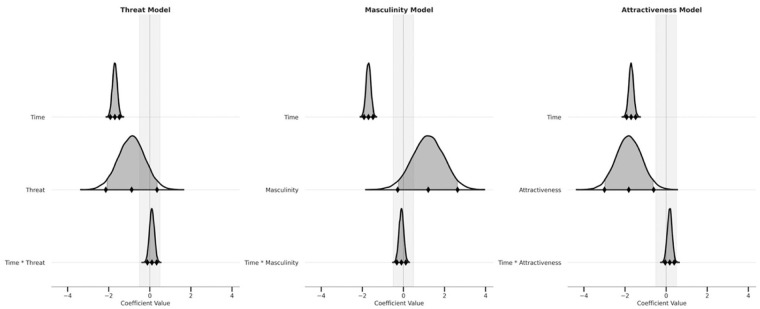
Posterior distributions of the effect of time, trait, and their interaction across all three models in Study Three. Shaded region indicates the ROPE of ±0.5% units, black diamonds indicate the median and lower and upper 95% quantiles.

Here, we use the Bayesian analyses described in Study Two to further understand the lack of evidence for the main effects and interactions. As described in Study 2, we compute the 95% posterior density interval, the probability of direction of effects (Makowski et al., 2019), as well as examining the amount of the posterior distribution that falls within the ROPE (Kruschke, 2018), which we set at ±0.50, or half a percentage point. We estimated these models using weakly informative, normally distributed priors on all the fixed and random effects. The results are illustrated in Figure 6, and with specific details in Table 2. As in Study 2, we focus the discussion on the main effects and interactions, given that the effect of Time in each model was identical to the frequentist estimate.

**Table 2.** Posterior Summaries for the Models Fit to Data in Study Three.

Rating	Parameter	Posterior Mean	Posterior SD	95% Lower	95% Upper	*p*(parameter| data) < 0	*p*(parameter| data) null
Threat	Time	−1.702	0.115	−1.922	−1.468	1	0
	Threat	−0.887	0.651	−2.088	0.473	0.914	0.259
	Time * Threat	0.105	0.118	−0.123	0.337	0.189	1
Masculinity	Time	−1.702	0.115	−1.919	−1.47	1	0
	Masculinity	1.196	0.766	−0.253	2.759	0.059	0.167
	Time * Masculinity	−0.099	0.114	−0.323	0.123	0.807	1
Attractiveness	Time	−1.701	0.115	−1.922	−1.469	1	0
	Attractiveness	−1.817	0.63	−3.033	−0.574	0.998	0.018
	Time * Attractiveness	0.172	0.115	−0.05	0.397	0.069	0.998

For the threat model, the estimate of the main effect of threat was almost identical to the frequentist model, *b* = −0.88. However, the probability that the effect was negative – in line with the hypothesis that increased threat led to less focus on faces – was 91%. While this supports the hypothesis, the magnitude of this effect is small, ranging from a decrease of 2% points (lower 95% interval) to a possibility of around 0.5% points (upper 95% interval). Fully a quarter of the posterior of this effect fell in the ROPE of between ±0.5% points, suggesting about a 1 in 4 chance this effect is practically null. For the interaction term, which suggests a differing focus on faces with increased viewing time, the posterior fell entirely within the ROPE, and so we accept the null for this parameter.

For masculinity, and much like in Study 2, the probability that the main effect is positive (a longer dwell time on heads of stimuli perceived as more masculine) was around 94%, with an upper bound of an increase of 2.76% points. Confirming the results of Study 2, the interaction coefficient fell entirely within the ROPE.

For attractiveness, we noted very different results from Study 2, with a 99.8% probability of the main effect of attractiveness being negative (increased attractiveness led to lower dwell times on faces). However, as in Study 2, the probability that the effect was entirely in the ROPE was 99.8%.

Taken together, these results suggested that the non-significant interactions in the main analysis are consistent with there being no effect – higher levels of the perceived trait are not associated with changes in dwell time with increased viewing time.

### Additional Exploratory Analysis – Pooling Across All Studies

As a final exploratory analysis, we combined all our individual datasets to further leverage the partial-pooling properties of hierarchical models. As before, we analyzed each trait separately, but this time we used a random-effects structure that allowed us to estimate the key time-by-trait rating interaction and its variability across studies. Partial-pooling allows the data in each dataset to inform, and be informed by, the data in other studies, and so the overall fixed effect is a more robust and realistic estimate of the effect. Each model contained the main effects of time, trait rating, and their interaction, as before, as well as a random intercept for both participants and targets. We now also included a random intercept for the *dataset* (four levels) and set the interaction term to be a random slope, such that variability in this effect across studies was accounted for and fed into the overall estimate of the effect. Table 3 summarizes the number of judges and targets for each part of the analysis.

**Table 3.** A summary of the Hypothesized and Observed Effects Present in the Current Data, Broken Down by the Study, Judge Sample Size, Target Sample Size, and Analysis Phase.

Phase	Sample		Model parameters			
	Judges	Targets	Time	Threat	Masculine	Attractive
Hypothesized effects			**Main effect: −**	**Main effect: +** Interaction: +	**Main effect: +** Interaction: +	**Main effect: +** Interaction: +
Study One						
Female only	27	12^female^	**Main effect: -**	**Main effect: +** Interaction: -	**Main effect: -** Interaction: +	**Main effect: -** Interaction: -
Study Two						
All targets	30	22^mixed^	**Main effect: -**	Main effect: 0 Interaction: 0	Main effect: 0 Interaction: 0	Main effect: 0 Interaction: 0
Female only	30	11^female^	**Main effect: -**	Main effect: 0 Interaction: 0	Main effect: 0 Interaction: 0	Main effect: 0 Interaction: 0
Male only	30	11^male^	**Main effect: -**	**Main effect: -** Interaction: 0	Main effect: 0 **Interaction: +**	Main effect: 0 Interaction: 0
Study Three						
All Targets	48	22^mixed^	**Main effect: -**	Main effect: 0 Interaction: 0	Main effect: 0 Interaction: 0	**Main effect: -** Interaction: 0
Female only	48	11^female^	**Main effect: -**	**Main effect: -** Interaction: +	**Main effect: -** Interaction: +	**Main effect: -** Interaction: 0
Male only	48	11^male^	**Main effect: -**	Main effect: 0 Interaction: 0	Main effect: 0 Interaction: 0	Main effect: 0 Interaction: 0
All Studies						
All Targets	105	22^mixed^	**Main effect: -**	Main effect: 0 Interaction: 0	**Main effect: +** Interaction: 0	**Main effect: -** Interaction: 0
Female only	105	11^female^	**Main effect: -**	Main effect: 0 Interaction: 0	Main effect: 0 Interaction: 0	**Main effect: -** Interaction: 0
Male only	78	11^male^	**Main effect: -**	Main effect: 0 Interaction: 0	Main effect: 0 Interaction: 0	Main effect: 0 Interaction: 0

*Note.* **Bold** text indicates a notable difference.

+ indicates a positive effect, − indicates a negative effect, 0 indicates no effect.

#### Male and Female Targets Together

##### Ratings of Threat

The pooled threat model showed a significant effect of time, *b* = −1.45, *SE* = 0.08, *t*(22067.85) = 16.84, *p* < .001, indicating that dwell time decreased by 1.45% points with each second of viewing time. There was no significant effect of threat, *b* = −0.02, *SE* = 0.50, *t*(20905.78) = 0.05, *p* = .964, nor evidence of an interaction, *b* = −0.14, *SE* = 0.10, *t*(12.731) = 1.39, *p* = .190.

##### Ratings of Masculinity

For masculinity, we observed a significant effect of time, *b* = −1.45, *SE* = 0.08, *t*(22068.64) = 16.84, *p* < .001, suggesting that dwell time decreased by 1.45% points with each second of viewing time. Contrary to the individual study analyses, there was a significant effect of masculinity, *b* = 1.83, *SE* = 0.54, *t*(2184.22) = 3.38, *p* = .001, indicating higher masculinity ratings resulted in more dwell time on the head. There was no evidence of an interaction, *b* = −0.21, *SE* = 0.18, *t*(3.82) = 1.13, *p* = .326.

##### Ratings of Attractiveness

Dwell time was also significant in this model *b* = −1.45, *SE* = 0.08, *t*(22067.85) = 16.84, *p* < .001, decreasing by 1.45% points with each second of viewing time. Again, in contrast to Study 2, there was a negative effect, a significant effect of attractiveness, *b* = −1.40, *SE* = 0.45, *t*(21545.36) = 2.99, *p* = .003 – increased attractiveness resulted in less dwell time on the head. Finally, there was no evidence of an interaction, *b* = 0.30, *SE* = 0.18, *t*(4.06) = 1.68, *p* = .168.

As before, we also estimated these models separately for male and female targets, which we report below:

#### Female Targets Only

##### Ratings of Threat

The pooled threat model for female targets only showed a significant effect of time, *b* = −1.35, *SE* = 0.12, *t*(12086.59) = 11.55, *p* < .001. There was no significant effect of threat, *b* = 0.07, *SE* = 0.72, *t*(12625.51) = 0.10, *p* = .992, nor an interaction *b* =−0.14, *SE* = 0.17, *t*(8.68) = 0.81, *p* = .441.

##### Ratings of Masculinity

For masculinity, we observed a significant effect of time, *b* = −1.31, *SE* = 0.15, *t*(8333.15) = 8.78, *p* < .001. There was a non-significant effect of masculinity, *b* = 1.56, *SE* = 0.82, *t*(12172.72) = 1.90, *p* = .058, nor an interaction, *b* = −0.14, *SE* = 0.27, t(4.77) = 0.53, *p* = .622.

##### Ratings of Attractiveness

Dwell time was also significant *b* = −1.36, *SE* = 0.11, *t*(3.37) = 9.99, *p* < .001, and a significant effect of attractiveness, *b* = −2.08, *SE* = 0.62, *t*(12422.86) = 3.30, *p* = .001 – increased attractiveness resulted in less dwell time on the head. There was no evidence of an interaction, *b* = 0.35, *SE* = 0.19, *t*(5.02) = 1.79, *p* = .133.

#### Male Targets Only

##### Ratings of Threat

The pooled threat model for male targets only showed a significant effect of time, *b* = −1.59, *SE* = 0.14, *t*(9355.01) = 11.50, *p* < .001. There was no significant effect of threat, *b* = −1.06. *SE* = 0.74, *t*(8337.44) = 1.44, *p* = .151, nor an interaction *b* = −0.05, *SE* = 0.18, *t*(4.99) = 0.24, *p* = .788.

##### Ratings of Masculinity

For masculinity, we observed a significant effect of time, *b* = −1.82, *SE* = 0.23, *t*(9357.61) = 7.85, *p* < .001. There was a non-significant effect of masculinity, *b* = 0.94, *SE* = 1.26, *t*(9236.94) = 0.74, *p* = .457, nor an interaction, *b* = 0.28, *SE* = 0.43, t(3.44) = 0.65, *p* = .559.

##### Ratings of Attractiveness

Dwell time was also significant *b* = −1.58, *SE* = 0.14, *t*(9351.12) = 11.61, *p* < .001, but there was a non-significant effect of attractiveness, *b* = −0.58, *SE* = 0.78, *t*(8965.66) = 0.76, *p* = .446. There was no evidence of an interaction, *b* = 0.04, *SE* = 0.20, *t*(4.65) = 0.21, *p* = .841.

##### Correlations Between Ratings

In an additional, requested, analysis, we analyzed the relationship between the three rated adjectives in the pooled data to investigate the independence of the ratings. We used a hierarchical model to simultaneously extract correlations between threat, masculinity, and attractiveness ratings. By nesting trait ratings within participants and targets, a random slope for each participant and target could be estimated, along with the correlations amongst them. While the by-participant correlations reflect the degree to which participants who, for example, give higher ratings of masculinity also give higher ratings of attractiveness, the by-target correlations indicate whether the trait ratings of targets move together systematically, and so we focus on these. These correlations suggest threat and masculinity are strongly positively correlated, *r* = .82, while attractiveness and masculinity are negatively associated, *r* = −.59. Similarly, threat and attractiveness are negatively correlated, *r* = −.63. Given the strength of these associations, it is worth noting that the pattern of rating effects was not consistent across studies and stimuli (see next section).

### All Studies’ Results at a Glance

In the analysis of these studies, we have tested data from three studies and with the targets together, and with male and female targets separately. Table 3 presents an overview of the hypothesized and observed findings. As an overall picture, the hypothesis that individuals would dwell less on the faces of targets over time was observed in all analyses.

Whilst Study One provided evidence to support hypotheses about how ratings may impact dwell time on the face, this was not in line with the hypothesized directions. Further, analysis of whole samples and female-only targets in the other two studies failed to replicate these findings. Analysis of male-only targets in Studies Two and Three provided no consistent pattern of effects.

In the most robust analysis of the whole-sample data, we observed that more masculine or less attractive targets had greater dwell time on the face. No interactions were observed.

## Discussion

Here, we report three studies: two exploratory and one confirmatory in a registered report. The line of research represents rigorous attempts to self-replicate findings to quality check the robustness of our claims. In our Study One, we found promising evidence that ratings of threat, masculinity, and attractiveness may reflect different observation patterns of approaching people. Namely, that attention to the face of targets may vary as a function of social perception. However, due to a small sample size of judges and a limited pool of female-only targets, we were not confident in these findings alone. We attempted to replicate and expand this study with a new sample in a new location, with the inclusion of male targets as well. This replication attempt failed, with none of the original hypothesised effects being present in this second sample. Faced with a first study that had results in line with our hypotheses and a second, more robust, study that did not find effects, we registered a third study. This study drew on the stronger methodology of the second sample and collected data from two sites for a robust confirmatory test. This study found more effects than the second study but the opposite effect to the first study. Overall, we consider this no consistent pattern of evidence from the individual studies for us to confidently support our hypotheses.

Using the whole data set from across the studies, we do find some evidence that perceived masculinity is associated with dwelling on the faces of unknown other people. Similarly, increased perceived attractiveness was associated with more time observing the whole body of the target. Notably, these more robust analyses on a large sample size (for eyetracking research norms: *N* = 105) were not a strong support of the Study One findings, which found an effect of threat not present in the whole sample, and the opposite effect of masculinity. We consider our research programme here a lesson in the utility of replication, registered reports, and treating one’s own findings with caution. Assuming our whole-sample analysis is the most robust, we observed individual study findings that were false-positives and false-negatives. These findings occurred when we would traditionally attempt to publish our results and may have given a different impression to the literature if not for our further confirmatory analysis.

### Eyetracking and Social Judgements

A review of the existing literature on person perception will find that the typical methodology focuses on presenting participants with static faces as cues to social judgements (see Satchell et al., 2023). Using stimuli that can be shared efficiently and are limited in their complexity enables powerful worldwide research (Jones, DeBruine, et al., 2021) but may be limited in terms of its ability to speak to the everyday experience of encountering new people (Satchell, 2019; Satchell et al., 2023). Here, we were interested in studying how observing full-body walking stimuli might affect our perceptions of unknown others. Others have addressed questions of full-body motion before (Gunns et al., 2002; Roether et al., 2009; Satchell et al., 2018, 2021), but we add to this literature by considering the ways in which a judge might attend to the different aspects of an approaching person. Like previous research (Azarian et al., 2016a; Eastwood et al., 2001; Garza et al., 2017; Gilbert et al., 2011; Rodway et al., 2019; Sidhu et al., 2021), we monitored participants’ gaze with an eyetracker. Specifically, to address all the above points, we were interested in how there might be dwell time on the target person’s face and how this might change over time. We found that it is the case that, even with full-body stimuli present, participants attended to the face of an unknown person first. Within the first second, participants typically dwelled on the face region of our targets. However, across all our studies, we saw this fixation duration decline over time. With even the short 10 s window of our stimuli, participants were much less likely to be dwelling on faces as the videos progressed. Further, we did find evidence that the perceived attractiveness and masculinity of a target affected this dwell time. Focusing on the face was more prevalent when a target was also rated as more masculine or less attractive. There was no robust evidence of an interaction whereby the social perception variables interacted with time to generate different observational styles.

These studies aimed to introduce a move towards more naturalistic presentations of stimuli in the experimental social perception literature. Since the initial conception of this paper, there has been new research using more everyday methodologies, also exploring how often individuals attend to faces. In their study of 33 participants walking around campus wearing wearable eyetracking equipment, Varela et al. (2023) found that their sample only fixated on faces 14% of the time. Their sample was following a known route through a busy University site, and in this typical experience, they spent limited time attending specifically to the faces of unknown others. Much like our current findings, these results suggest that faces are initially interesting but not the sole focus of our attention to new people. We would consider our current findings to add to the rationale of future work to more regularly use whole-body, dynamic, stimuli. It is a limitation of the current study that our participants sat on a fixed eyetracker and not able to socially explore, like the research by Varela et al. (2023). Future research should bring together social judgement research with ‘in the wild’ dynamic eyetracking. Perhaps by asking participants to review the video-only footage of a walk through a busy social setting, and ask them to make social judgement ratings. These could then be linked to the eyetracking to study differing observation styles depending on social perception.

Similarly, we would encourage more research to integrate social perception and social attention research. We find differing attention to the faces and bodies of targets depending on their perceived attractiveness or masculinity. It is of interest to understand how the individual’s whole body might solicit attention in different ways depending on their perceived social value or even the contextual value. Fashion, hairstyles, and accessories all solicit social attention and impact social judgements in different ways (i.e. Sidhu et al., 2021), and understanding this through eyetracking would enhance social psychology, as well as interdisciplinary collaborations with researchers in the arts. It is a limitation of the current study that our sample was dressed in a standardized presentation (white t-shirt, dark shorts), and it would be interesting to learn more about how more natural presentations of clothing impact hold our attention and affect our perceptions of others.

### Interpreting Inconsistent Findings

A reasonable question about the current findings is the extent to which the differences between the studies might explain differences in the results. Methodologically, these three studies are highly similar and reasonably should not differ in terms of their psychological experience. However, this perhaps minimizes the differences between Study One and Studies Two and Three. In Study One, there were only female targets, and it found the most expected effects. The later studies effectively doubled (from *k* = 12 to *k* = 22) the total number of stimuli by including male targets. Perhaps, there might be a context effect whereby the presence of male stimuli in the array changed the way the female targets were perceived. To know more about whether this drove the differences between studies, perhaps some future research might look at the difference between samples of stimuli as context. Further, in a limitation of our study, our targets were relatively homogenous in age and demographics (other than gender), and so understanding the contextual impacts of rating a more diverse selection of stimuli might be of interest. Of course, if the homogeneity of the stimuli in Study One is the mechanism that induced the observed effects, then those findings are not particularly interesting in terms of their application. In an everyday context, we observe a range of individuals who differ on a whole range of features. If one can only elicit the threat effects when presented with limited stimuli, then it has limited utility for theory building and application.

On reviewing our findings, we consider the most reasonable explanation for our pattern of results to be that the variability we observed is due to small (albeit eyetracking study typical) sample sizes of judges. Even with the large statistical power of our high number of cases of data (analysing variability over 10 s × 12–22 stimuli × 78–108 judges), our individual study conclusions are still based on ratings of 27 to 48 judges alone. Inconsistent patterns of findings from sample sizes of this size might not be a surprise. We should expect varying false-positive and false-negative effects when the sample sizes generating the data are smaller. It is for this reason we draw on our whole-sample aggregate analysis to draw reasonable conclusions from this project and would be cautious of over-interpreting differences between the studies as theoretically meaningful.

### Constraints of Context

The nature of the laboratory experiment introduces constraints on participants’ ability to respond to the study. Not only is this a consequence of the limited mobility of our participants, who are using a stationary eyetracker (as discussed above), but also in the ways in which they can respond to the study. As noted elsewhere (Satchell et al., 2023), it is a limitation of much of the social judgement literature that participants can only reply to provided key words in a fixed approach. We asked our sample to specifically consider threat, masculinity and attractiveness. This may not be the core considerations that individuals might have in an everyday context. Recent research has pointed to the benefits of using free-responding and how participants’ open text data might be analyzed reproducibly (Jones et al., 2024). It would be of interest in future research to not lead our participants as much as we might enable them to respond with their own free descriptions of unknown others.

Relatedly, participants’ responses and even eye movements are shaped by the context of social observation. The approach to social judgement and the behaviours used to understand new others will be motivated by the intended outcomes of the judges. Here, our observation was not given a purpose or context. Participants were simply asked to view videos of people walking. Research has shown that instructions to simply view videos leads to different eyetracking behaviours, so much so that there are no differences between people diagnosed with autism spectrum disorders or not when they are socially attending to others (Kikuchi et al., 2022) or told they are watching live (as opposed to pre-recorded) video (López et al., 2023), in stark contrast to the differences in photograph and video alone research. We can see reasonable arguments that eyetracking patterns might also differ if someone is assessing a potential person arriving for a job interview or a first date, or approaching at night in the dark. Future research should consider these potential impacts of goal-orientation on eyetracking and social judgement consequences.

## Conclusion

The current paper presents the results of inconsistent individual studies investigating how attention to the face of an approaching unknown person may change over time and be related to our social judgements of others. Aggregate analysis suggests that individuals look less at the face over the first few seconds of a social interaction, and that more feminine and attractive-looking people might receive more full-body observations. This paper also provides an insight into the risks of false-positive and false-negative research findings and uses a registered report to deliver a replication to conclude an inconsistent pattern of findings. As such, this work is a note of caution for experimental task research and encourages future research to follow the review process undertaken here.
